# Medium-term outcomes after robotic-assisted lateral suspension with mesh for advanced multi-compartmental prolapse

**DOI:** 10.1007/s00192-019-04069-7

**Published:** 2019-08-06

**Authors:** Eleonora Russo, Andrea Giannini, Magdalena Montt Guevara, Paolo Mannella, Giulia Misasi, Maria Falcone, Tommaso Simoncini

**Affiliations:** Obstetrics and Gynecology, Department of Clinical and Experimental Medicine, University of Pisa, Via Roma, 67, 56126 Pisa, Italy

**Keywords:** Abdominal lateral suspension, Anterior defect, Apical defect, Multi-compartmental pelvic organ prolapse (POP), Robotic surgery

## Abstract

**Introduction and hypothesis:**

Robotic abdominal lateral suspension (RALS) is an innovative mini-invasive surgical technique that allows treating apical and anterior prolapse. The safety and efficacy of this strategy have not yet been tested.

**Methods:**

We completed a prospective case series of 115 RALS to treat apical and anterior prolapse stage III or IV, with no or minimal (stage I) posterior defect. Clinical evaluation was performed with a simplified POP quantification system (POP-Q). Mean follow-up was 28 ± 4 months. Primary outcomes were objective and subjective cure; secondary outcomes were reoperation rate for recurrence, erosion rate and complications. Objective cure was defined as POP-Q ≤ 1. Subjective cure was defined as absence of vaginal bulge. Patient’s satisfaction was measured using the Patient Global Impression of Improvement Scale (PGI-I).

**Results:**

There was a significant improvement in POP-Q score in all treated compartments with an objective cure rate of 88.7% for the anterior and 93.1% for the apical compartment (*p* < 0.0001). Subjective cure rate was 82%. The emergence of de novo high rectoceles was not significant in the cohort, as much as the development of de novo stress or urge urinary incontinence. Reoperation rate for POP was 11.3% (8 recurrent cystoceles without apical descent and 5 apical and anterior relapses). No postoperative complications of Clavien-Dindo grade ≥ 3a were seen. Mesh exposure rate was 0.9%; 58.2% patients compiled a PGI-I score at 18–24 months post-surgery, reporting high satisfaction rates.

**Conclusions:**

RALS is highly effective at a mid-term follow-up for the treatment of advanced apical and anterior POP.

## Introduction

Pelvic organ prolapse (POP) is a major health problem affecting up to 50% of women, and the prevalence increases with age [1]. Apical prolapse is defined as the descent of the cervix or vaginal cuff after hysterectomy [2]. The correction of apical POP represents one of the major challenges in reconstructive pelvic floor surgery.

Many surgical procedures exist, including vaginal or abdominal approaches. There is an ongoing debate about which procedure represents the most effective, safe and durable option [3, 4].

Sacrocolpopexy (SC) is considered the reference standard for apical POP. SC offers better postoperative results than vaginal surgical techniques [4]. Laparoscopic sacrocolpopexy (LSC) and more recently robotic-assisted sacrocolpopexy (RASC) have been developed as minimally invasive adaptations of SC. Sacrocolpopexy may be associated with rare but potentially serious morbidity, as dissection at the level of the sacral promontory can hinder potentially lethal vascular complications, particularly in obese women or in the presence of vascular anatomical variations. Moreover, intraoperative complications include intervertebral discitis and ureteral and nerve injuries.

Abdominal lateral suspension (ALS) with mesh is an alternative strategy to restore apical prolapse that does not require dissection of the sacral promontory. This technique was described first by Kapandji in 1967 and by Cornier and Madelenat in 1994 [5, 6]. The procedure has subsequently been modified and adapted to laparoscopy by Dubuisson [7]. ALS is performed with a T-shaped synthetic mesh graft placed in the vesico-vaginal septum and sutured to the anterior vaginal wall, uterine cervix and isthmus. The lateral arms of the mesh are suspended bilaterally to the abdominal wall, posterior to the anterior superior iliac spine. The procedure allows treating concomitantly apical and anterior POP.

Published data on laparoscopic lateral suspension (LLS) show an objective success rate of > 90% after 1 year on both the anterior and apical compartment, comparable to LSC [8–11].

Robotic assistance is perceived by many surgeons to offer specific advantages in pelvic floor reconstructive procedures. The robotic platform allows a more ergonomic environment that simplifies complex laparoscopic tasks such as suturing, knot tying and pre-sacral dissection; however, an advantage over traditional laparoscopy on surgical outcomes has not yet been demonstrated. Nonetheless, the overall cure rate of apical prolapse reported for RASC ranges between 97 and 100% in the available studies [12, 13].

Currently, there are limited published retrospective and no prospective trials on robotic lateral suspension (RALS) [14, 15]. In 2016, we described the surgical technique and the short-term outcomes on our first 40 consecutive RALS procedures [15].

We now report the surgical and clinical outcomes of a prospective cohort of 115 consecutive patients treated with RALS for advanced anterior and apical POP, with a minimum follow-up of 2 years.

## Materials and methods

This is a prospective case series of 115 consecutive patients who underwent RALS for symptomatic advanced anterior and apical POP between September 2014 and January 2017.

A complete clinical assessment was performed after 6, 12 and > 24 months.

The surgical procedures were performed by the same surgeon at the Pisa University Hospital as previously described [15].

POP was defined according to the pelvic organ prolapse quantification (POP-Q) system and evaluated in lithotomic position during a Valsalva maneuver. A simplified POP-Q with three points (points Ba, Bp, C) was used [16].

Inclusion criteria were symptomatic apical and anterior prolapse stage ≥ III, negative cervical cytology and no abnormal uterine bleeding. The concomitant presence of urinary incontinence was not considered an exclusion criterion. Patients with enterocele and/or high rectocele POP-Q stage > I were excluded. Concomitant surgical procedures were performed in a few cases.

Preoperative demographic characteristics, POP-Q stage and prolapse-related symptoms were documented. The postoperative examination included POP-Q stage measurement and targeted questions about subjective satisfaction.

Surgical outcomes were reported following the International Urogynecological Association recommendations [17]. Main outcome measures were subjective and objective cure at a mean follow-up of 2.4 years. Anatomic objective cure was defined satisfactory as POP-Q ≤ 1. Subjective cure was defined as absence of vaginal bulge as indicated by a negative response to the question “Do you usually have a sensation of bulging or protrusion from the vaginal area?” In addition, patient satisfaction was evaluated using the Patient Global Impression of improvement Scale (PGI-I) through a telephone interview performed 18–24 months after index surgery [18].

Secondary outcomes were reoperation rate for symptomatic POP, erosion rate, complications and postoperative incidence of lower urinary tract symptoms (LUTS). Complications were evaluated with the Clavien-Dindo classification and classified using the joint International Urogynecological Association/International Continence Society (IUGA/ICS) complication classification [19, 20].

This study was carried out in accordance with the recommendations of the Good Clinical Practice (ICH/GCP), Ministerial Decree of 1997. The protocol was approved by the Regional Ethics Committee for Clinical Trials, Tuscan North West Wide Area. All subjects gave written informed consent in accordance with the Declaration of Helsinki.

Statistical analysis was performed using GraphPad Prism 7 (GraphPad Software). Categorical variables were presented with percentages; continuous variables were presented with means and standard deviations. Shapiro-Wilk normality test was used to determine the normality of data distribution. In accordance, Wilcoxon matched-pairs signed-rank one-tail test was used to study pre- and postoperative outcomes. In addition, Kruskal-Wallis test was performed followed by Dunn’s multiple comparisons test to analyze the outcomes among 0, 6 and 12 months after surgery. The values of *p* < 0.05 were considered significant (*p < 0.05; **p < 0.01; ***p < 0.001).

## Results

### Patient characteristics and perioperative outcomes

We included 115 consecutive robotic-assisted lateral suspensions for symptomatic advanced (stage III–IV) apical and anterior prolapse, with no (*N* = 106) or minimal (stage I, *N* = 9) high posterior defects. Preoperative POP symptoms were vaginal bulge and obstructed voiding. Demographic data, surgical history and POP-related symptoms are summarized in Table 1.

**Table 1:** Preoperative demographic characteristics

Age (years), mean ± SD	64.6 ± 8.5
Nulliparous, *n* (%)	1 (0.9%)
BMI (kg/m^2^), mean ± SD	26.6 ± 4.6
Menopausal, *n* (%)	107 (93%)
Prior hysterectomy, *n* (%)	9 (7.8%)
Prior POP surgery	
Abdominal POP surgery, *n* (%)	1 (0.8%)
Vaginal POP surgery, *n* (%)	9 (6.9%)
Prior stress urinary incontinence surgery	
None, *n* (%)	113 (98.2%)
Transobturator sub-urethral sling, *n* (%)	2 (1.7%)
POP-Q at baseline	
Point Ba ≥ − 1, *n* (%)	111 (96.5%)
Point C ≥ −1, *n* (%)	115 (100%)
Point Bp ≥ −1, *n* (%)	9 (7.8%)
Occult stress urinary incontinence, *n* (%)	18 (15.6%)
Urinary urgency, *n* (%)	19 (16.5%)
Fecal Incontinence, *n* (%)	4 (3.4%)
Vaginal bulge, *n* (%)	115 (100%)

Data are presented as number of cases (*n*), percentages (%) or median ± SD

*POP* pelvic organ prolapse, *POP-Q* Pelvic Organ Prolapse Quantification System (simplified)

In 103 patients the uterus was preserved, while in 3 patients a supracervical hysterectomy was performed because of fibromatosis. In nine patients RALS was performed on the vaginal vault. Twelve patients had concomitant procedures: stapled transanal rectal resection (STARR) (*N* = 1), cervical amputation (*N* = 1), ovarian cyst removal (*N* = 1) and bilateral adnexectomy (*N* = 9).

The mean operating time was 129 ± 34 min, the mean blood loss was 50 ml, and the mean postoperative hospitalization stay was 1 day. There were no conversions to laparotomy or intraoperative complications.

### Anatomic and functional outcomes

Anatomic outcomes are shown in Tables 2 and 3. The mean postoperative follow-up was 28 ± 4 months. There was a significant improvement in POP-Q score in all treated compartments with an overall objective cure rate of 88.3% for the anterior compartment and 93.1% for the apical compartment.

**Table 2:** Anatomical outcomes based on clinical evaluation with a simplified POP-Q (Pelvic Organ Prolapse Quantification System) measurement as defined by IUGA-ICS prolapse staging after a medium follow-up of 28 ± 4 months

	Prior surgery	After surgery (follow-up 28 ± 4 months)
POP-Q point Ba	3.31 ± 1.36	−2.34 ± 1.64***
POP-Q point C	4.09 ± 0.9	−6.47 ± 1.83***
POP-Q point Bp	−2.6 ± 1.15	−2.23 ± 1.18 *ns*†

Data are presented as median ± SD. Data after surgery include de novo prolapses (†). To identify the differences between pre- and postoperative outcomes, Wilcoxon matched-pairs signed-rank one-tail test was performed (***p < 0.001 versus prior surgery); *ns*, not significant

**Table 3:** Recurrence of prolapse after RALS at 6, 12 and 24 months based on clinical evaluation with a simplified POP-Q (Pelvic Organ Prolapse Quantification System) measurement

	Prior surgery	After surgery		
		6 months	12 months	24 months
POP-Q point Ba ≥ −1	111 (96.1%)	8 (8.2%)***	4 (3.6%)***	1 (1%)***
POP-Q point C ≥ −1	115 (100%)	7 (6%)***	1 (0.9%)***	0***

Data after surgery include de novo prolapse (†). Data are presented as number of cases (*n*) and percentages (%). To analyze the outcomes among 0, 6, 12 and 24 months, Kruskal-Wallis test followed by Dunn’s multiple comparisons test was performed (***p < 0.001 versus prior surgery)

According to the pre-set boundaries for relapse, an anatomical failure developed in 17 patients (14.7%) and a second surgery was needed in 13 cases (11.3%). Most relapses developed within the first 6 months after surgery (Table 3). Eight cases (6.9%) had an isolated recurrence in the anterior compartment. All these patients developed a cystocele that was caudal to the anterior flap of the mesh, which otherwise remained well suspended and anchored to the anterior vaginal wall and to the cervix. All these patients were corrected with anterior colporraphy and did well thereafter.

Five patients (4.3%) had a symptomatic combined apical and anterior relapse due to sliding of the lateral arms. They were all re-operated abdominally: in two patients a laparoscopic re-suspension of the lateral arms was possible, while in the other three patients a sacral suspension after removal of the lateral arms mesh was performed. All the patients did well afterward. Three patients developed an isolated and asymptomatic second-stage apical prolapse, but they did not require a re-surgery. Interestingly, two patients showed a cervical elongation at follow-up, but we did not have the information on cervical length available before surgery, so it is unclear whether the elongation was present at baseline. Both patients were however asymptomatic. The overall reoperation rate is shown in Table 4.

**Table 4:** Record of additional surgery

Repeat surgery for recurrence *n* (%)	
Robot-assisted sacrocolpopexy	2 (1.7%)
Laparotomic sacrocolpopexy	1 (0.9%)
Laparoscopic lateral mesh re-suspension	2 (1.7%)
Anterior colporraphy	8 (6.9%)
Primary POP surgery/different site *n* (%)	
Laparoscopic ventral rectopexy	1 (0.9%)
Surgery for complications *n* (%)	
Lateral arm mobilization	2 (1.7%)
Vaginal mesh erosion removal	1 (0.9%)

Includes surgery for recurrent symptomatic pelvic organ prolapse (POP), surgery for de novo POP and surgery for complications. Data are presented as number of cases (*n*), percentages (%)

Nine patients (7.8%) had a high rectocele POP-Q stage I at enrollment. In seven patients the rectocele disappeared after surgery. The other two patients had persistence of the high posterior defect. In the rest of the cohort, where no posterior defect was present at enrollment, seven (6%) women developed a de novo high rectocele POP-Q stage I-II, all of which were asymptomatic.

Regarding urinary symptoms, no systematic urodynamic preoperative investigation was performed. Before surgery, 18 women (15.6%) had occult stress urinary incontinence (SUI) during clinical examination. All these patients remained continent after surgery.

De novo SUI occurred in four patients (3.4%) and a mid-urethral sling was inserted in three cases.

Nineteen patients (16.5%) had urinary urgency without incontinence before surgery. In 18 patients urgency disappeared after lateral suspension, possibly linked to the resolution of the anterior defect. No patient developed de novo urge symptoms after lateral suspension.

Two patients experienced voiding dysfunction within 1 year in the absence of POP recurrence. In both cases the voiding dysfunction was associated with a post-voiding residual > 150 ml. One patient developed obstructed defecation symptoms. No patient developed fecal incontinence or dyspareunia.

After 2 years the subjective cure rate, in terms of absence of perceived vaginal bulge, was 88.7%.

## Postoperative complications

No patient had major postoperative complications (Clavien-Dindo grade ≥ 3a). Six patients experienced postoperative pain at the level of the lateral mesh arm suspension (Clavien-Dindo I). Two patients required mesh mobilization while the remaining four patients experienced spontaneous pain relief after 3 months. In both cases where surgery was needed, the pain was at the level of the fascia at the sites where the mesh reached the abdominal wall. A small superficial incision was made, and the mesh was isolated and freed from the attachment on the fascia. In both cases immediate relief from pain was obtained. However, three patients had pain due to a mono-lateral hematoma.

One patient had anterior vaginal wall mesh exposure: the vaginal exposure was grade 1 (exposure < 1 cm); the mesh complication occurred within 2 months of surgery and was located away from the suture lines, classified as 2AT2S1, according to the IUGA/ICS Prosthesis/Graft Complication Classification System. This complication required vaginal revision with partial mesh excision.

## Assessment of patient satisfaction

Sixty-seven patients (58.2%) participated in a telephone interview conducted in October 2018 when all the patients were 18–24 months post-surgery; 74.5% considered themselves better and 65.7% “much better or better” than before surgery based on the PGI-I scale. Results of telephone interviews are shown in Table 5.

**Table 5:** Patients' telephone interview results for the Global Impression of Improvement (PGI-I) scale

PGI-I scale rating	
Very much better	14 (20.9)
Much better	30 (44.7)
A little better	6 (8.9)
No change	6 (8.9)
A little worse	10 (14.9)
Much worse	1 (1.4)
Very much worse	0 (0)
No data	48 (41.7)

## Discussion

Our prospective case series shows that RALS is safe and highly effective in treating advanced apical and anterior prolapse at a medium-term follow-up. We observed a significant improvement in POP-Q score in all treated compartments with an overall objective cure rate of 88.3% for the anterior compartment and 93.1% for the apical compartment. After 28 ± 4 months, subjective cure rate, in terms of absence of perceived vaginal bulge, was 82%.

Abdominal surgery with mesh augmentation is a suitable approach to treat advanced and multi-compartmental prolapse. In the OPTIMAL trial, native tissue repair including apical suspension to the utero-sacral ligaments or the sacrospinous ligaments resulted in 60–70% surgical failure after 5 years from index surgery, which is significantly worse compared with the outcomes of sacral suspension [3, 21].

However, sacral suspension is a challenging procedure requiring advanced surgical skills and is also relatively inefficient in treating the anterior compartment [3]. The main advantage of lateral suspension over sacral suspension is the avoidance of sacral dissection with the related complications [22]. Recent anatomic studies on cadavers support the theory that the posterior displacement of the apex obtained with ASC limits the ability to correct advanced cystocele and favors anterior relapse [23]. In this view, lateral suspension may be the reference procedure to treat a combined anterior and apical defect. The peculiar shape of the mesh allows an effective restoration of the pubo-cervical fascia defect, and the lateral rather than posterior suspension is more effective in reducing anterior prolapse, while it does not address posterior prolapse. Therefore, lateral suspension and sacral suspension may have different surgical indications.

This is why we wanted to assess the possible role of lateral suspension in treating advanced apical and anterior prolapse, aiming to address the following key questions:

1. Is lateral suspension as effective as sacral suspension in restoring advanced apical prolapse?
2. Is lateral suspension as effective (or more effective) as sacral suspension in treating advanced anterior prolapse?
3. Is lateral suspension performed in patients without or with minimal high posterior prolapse promoting the development of a de novo posterior prolapse?

Our series addresses the first question satisfactorily, showing a cure rate for apical prolapse of 93.1%, which is comparable to the available series on robotic-assisted sacrocolpopexy and to the previously published results on laparoscopic lateral suspension (LLS) [9, 24].

Concerning the second question related to the anterior compartment, the anatomic failure in our series was 11.3%, which is in line with the results of LLS [24]. However, the published cohort on LLS reports included > 90% of patients with anterior POP ≤ stage III [24], while we systematically treated patients with POP-Q stage III and IV, thus with much higher risk of relapse.

With regard to the question on the posterior compartment, fair evidence suggests that the posterior direction of the vaginal axis and the mesh reinforcement of the recto-vaginal space achieved with sacrocolpopexy may improve bowel-related pelvic floor symptoms and posterior vaginal support in women with posterior and apical POP [25], thus making this procedure the gold standard to treat multi-compartmental prolapses. Lateral suspension does not address posterior prolapse, plus, there is a lingering hypothesis that if administered to patients without high rectocele, the lateral suspension of the apex may facilitate a later development of an enterocele or the descent of the upper part of the rectum [7, 8, 11].

In our cohort, we included a small group of patients with mild high posterior defects (stage I high rectocele) to investigate whether the lateral apical suspension may be effective in restoring high posterior defects or favor the emergence of high posterior defects in the long term. Surprisingly, our data show that most mild high rectoceles are treated effectively with lateral suspension of the apex. In addition, a de novo posterior defect is seen only in a minority (6%) of the patients after lateral suspension. This reinforces the concept that restoring level I support is important for both the apical and the posterior defect [26]. The rate of de novo enterocele after RALS in our series does not suggest the need for prophylactic treatment of the posterior compartment in the absence of preoperative enterocele or high rectocele. We believe that in the presence of such conditions patients submitted to RALS should also receive a concomitant transvaginal or transrectal posterior correction, or should be more appropriately treated with ASC.

Following lateral suspension, we observed two different types of relapses. The most frequent condition was the development of a cystocele that happened in patients where the mesh remained well attached to the lateral abdominal wall and to the apex, thus in the absence of an apical failure. In these patients the length of the anterior flap of the mesh did not entirely cover the vesico-vaginal space, and a distal cystocele developed, which was easily addressed transvaginally with anterior colporraphy. In a smaller percentage of cases we saw a sliding of the lateral arms of the mesh from the abdominal wall, leading to a combined apical and anterior relapse. We never observed a detachment of the mesh from the vagina or from the cervix based on US and abdominal exploration at re-do surgery. These cases were successfully treated with a second abdominal approach, with either a re-suspension of the lateral arms or conversion to sacral suspension. We believe this is useful information, as it shows that lateral suspension allows for a successful back-up surgery if needed.

Our procedures were all performed with robotic assistance. Whether robotics adds quality to POP surgery is an open question. Multiple levels of evidence support the efficacy and safety of RASC. Robotic sacrocolpopexy has good short- to medium-term results with few intra- and postoperative complications [12, 27, 28]. However, there is still a paucity of long-term data assessing the durability of robotic POP repair, and the available studies have significant methodological differences.

Based on our personal experience with the laparoscopic approach, robotic assistance may enhance the lateral suspension procedure particularly in two critical steps: the precise and deep dissection of the vesico-vaginal space and the mesh placement and fixation. Whether this leads to a more effective reconstruction surgery cannot be said, since we do not have a comparator laparoscopic group. However, we observed recurrence and reoperation rates similar to those described for laparoscopic or robotic sacral suspension (5–20%) in patients with advanced or recurrent prolapses [29]. In addition, our recurrence rates are similar to those published in a broad series of patients with significantly milder prolapses, where a laparoscopic lateral suspension was performed [7, 11, 24]. This may be an indirect suggestion that robotic assistance may improve the efficacy of lateral suspension compared with conventional laparoscopy and warrants future comparator studies. If this turns out to be true, robotic assistance may have a specific role in the selected group of patients with POP-Q stage ≥ III.

The incidence of postoperative complications is very low in our series. No severe postoperative complications (Clavien-Dindo grade ≥ 3a) occurred. Conversion to laparotomy was not necessary in any of our patients. This is similar to data reported for the laparoscopic lateral suspension procedure series [8, 9]. Notably, the overall postoperative complication rate reported for RASC is 11% [12], supporting the concept that in the absence of posterior prolapse, lateral suspension should be preferred over sacral suspension because of safety issues and better anterior correction.

Over more than 2 years of follow-up, we observed only one (early) mesh-related complication (0.8%), with a small mesh exposure, consistent with the data reported for other robotic-assisted or laparoscopic POP repair procedures [8, 14, 30, 31]. In all cases we used a T-shaped titanium-coated polypropylene mesh (TiLOOP®). Long-term absorbable sutures (Polydioxanone) were used to anchor the mesh to the anterior vaginal wall and non-absorbable 2-0 polypropylene stiches to anchor the mesh to the cervix. Polypropylene titanization results in a more hydrophilic material, which allows an easier mesh manipulation and fashioning and enhances mesh biocompatibility. We do not know whether the very low rate of mesh-related complications in our study is to be attributed to the specific coating of the mesh, to the use of monofilament sutures or to the precise robotic dissection and suturing. However, this stands in favor of the safety of robotic-assisted lateral suspension performed according to our described technique [15].

Related to the subjective experience of the patients, it is interesting to find that RALS in patients with clinically relevant bulging and LUTS, including voiding dysfunction, did not elicit worsening of the urinary symptoms or the emergence of stress or urge urinary incontinence. Rather, most patients with preoperative urgency had relief after RALS.

Only 58% of the cohort participated in the PGI-I interview. Seventy-five percent of the patients reported feeling better subjectively. This is slightly less than what was found at the clinical evaluations, where > 80% of the patients declared them free of vaginal bulge complaints. These discrepancies are frequent when comparing results of telephone interviews with in-person interviews. Of note, in the OPTIMAL trial, where native tissue repair was performed, only 60–65% of patients declared feeling better 3 years after surgery [21].

## Limitations of the study

Our study has some limitations. First, it was a single-center prospective case series that did not allow making inferences about the potential superiority of robotic lateral suspension versus laparoscopic lateral suspension or about the possible comparative efficacy of lateral versus sacral suspension. Furthermore, the assessment of storage and voiding urinary symptoms before and after surgery was not methodical; thus, we missed significant information on these aspects. Lastly, subjective outcomes were not investigated with a specific quality of life questionnaire.

## Conclusions

Surgical correction of advanced prolapse with robotic lateral suspension is a safe and highly effective technique and a feasible alternative to sacropexy in patients without posterior prolapse. Future prospective randomized studies are needed to better determine the long-term efficacy, morbidity and patient satisfaction for robotic lateral suspension and to compare this technique with sacropexy or the laparoscopic approach.
